# Symptom burden in community-dwelling older people with multimorbidity: a cross-sectional study

**DOI:** 10.1186/1471-2318-15-1

**Published:** 2015-01-05

**Authors:** Jeanette Eckerblad, Kersti Theander, Anne Ekdahl, Mitra Unosson, Ann-Britt Wirehn, Anna Milberg, Barbro Krevers, Tiny Jaarsma

**Affiliations:** Department of Social and Welfare Studies, Linkoping University, Linkoping, Sweden; Faculty of Health, Science and Technology, Department of Health Sciences, Nursing, Karlstad University, Karlstad, Sweden; Department of Geriatric Medicine and Department of Social and Welfare Studies, Linkoping University, Norrkoping, Sweden; Department of Neurobiology, Care Sciences and Society (NVS), Division of Clinical geriatrics, Karolinska Institutet (KI), Solna, Sweden; Local Health Care Research and Development Unit, County Council in Ostergotland, Linkoping University, Linkoping, Sweden; Department of Advanced Home Care and Department of Social and Welfare Studies, Linkoping University, Norrkoping, Sweden; Palliative Education & Research Centre, and Department of Social and Welfare Studies, Linkoping University, Norrkoping, Sweden; Department of Medicine and Health Sciences, Linkoping University, Linkoping, Sweden

**Keywords:** Chronic disease, Older people, Symptom assessment

## Abstract

**Background:**

Globally, the population is ageing and lives with several chronic diseases for decades. A high symptom burden is associated with a high use of healthcare, admissions to nursing homes, and reduced quality of life. The aims of this study were to describe the multidimensional symptom profile and symptom burden in community-dwelling older people with multimorbidity, and to describe factors related to symptom burden.

**Methods:**

A cross-sectional study including 378 community-dwelling people ≥ 75 years, who had been hospitalized ≥ 3 times during the previous year, had ≥ 3 diagnoses in their medical records. The Memorial Symptom Assessment Scale was used to assess the prevalence, frequency, severity, distress and symptom burden of 31 symptoms. A multiple linear regression was performed to identify factors related to total symptom burden.

**Results:**

The mean number of symptoms per participant was 8.5 (4.6), and the mean total symptom burden score was 0.62 (0.41). Pain was the symptom with the highest prevalence, frequency, severity and distress. Half of the study group reported the prevalence of lack of energy and a dry mouth. Poor vision, likelihood of depression, and diagnoses of the digestive system were independently related to the total symptom burden score.

**Conclusion:**

The older community-dwelling people with multimorbidity in this study suffered from a high symptom burden with a high prevalence of pain. Persons with poor vision, likelihood of depression, and diseases of the digestive system are at risk of a higher total symptom burden and might need age-specific standardized guidelines for appropriate management.

## Background

Chronic conditions among older people represent some of the largest health care challenges of this century, and one which will affect both the socioeconomics and the health care system [1]. Globally, the population is ageing and people can now live with several chronic diseases for decades [2]. Chronic diseases tend to increase with old age. An international systematic review reported that approximately 62% of all the people aged between 65–74 years, and 81.5% of people above 85 years suffer from multiple chronic diseases [3]. A co-occurrence of ≥2 diseases, where at least one is chronic, is defined as multimorbidity [4]. Multimorbidity is a condition with a high impact on functional impairment and quality of life [5, 6], and is a condition that often results in a variety of different symptoms [7].

In a number of studies, older people have been reported to suffer from various symptoms. These reports have been based on measurements of a single symptom such as depression [8] fatigue [9], sleep disorder [10] or pain [11]. Other studies have been based on only one symptom dimension, usually intensity or severity [12, 13]. The use of a multidimensional approach with the aforementioned dimensions is advocated both in research [14] and clinical practice [15]. A multidimensional assessment may also serve as a sufficient patient-reported outcome and it has been shown to be a sensitive tool to measure the effectiveness of interventions [16]. Symptom burden is a complex concept that goes beyond the scoring of any symptom instrument. To persons suffering from an advanced disease the impact of symptoms is often a well-known [17]. In an older population, high symptom burden is associated with increased health care utilization, frequent visits to the emergency department, hospitalization [18], admissions to nursing homes [19], and reduced quality of life [20, 21]. Symptom burden has been defined in different ways. In this study symptom burden is defined as "the subjective, quantifiable prevalence, frequency, and severity of symptoms placing a physiological burden on patients and producing multiple negative, physical, and emotional patient responses" [22].

Earlier studies within the research field of multidimensional symptoms have usually been conducted on specific chronic diseases, for example chronic obstructive pulmonary disease (COPD), heart failure (HF) or renal disease [20, 21, 23], and there is a paucity of studies focusing on multidimensional symptoms in older persons with multimorbidity. Existing guidelines for disease management are disease-specific, and they are not designed for people with multimorbidity [24]. If we set out to reduce the impact of diseases by reducing the symptom frequency, minimizing symptom severity, and relieving symptom distress it is essential to look at and assess the total symptom burden, not just one disease at the time.

### Aims

The aims of this study were to describe the multidimensional symptom profile and symptom burden of community-dwelling older people with multimorbidity. An additional aim was to describe which factors are related to symptom burden.

## Methods

### Design

This is a cross-sectional study using baseline data from a randomized controlled trial intended to include community-dwelling older people with multimorbidity and who had a great need for health care during the preceding year [25].

### Participants

**Figure 1:**
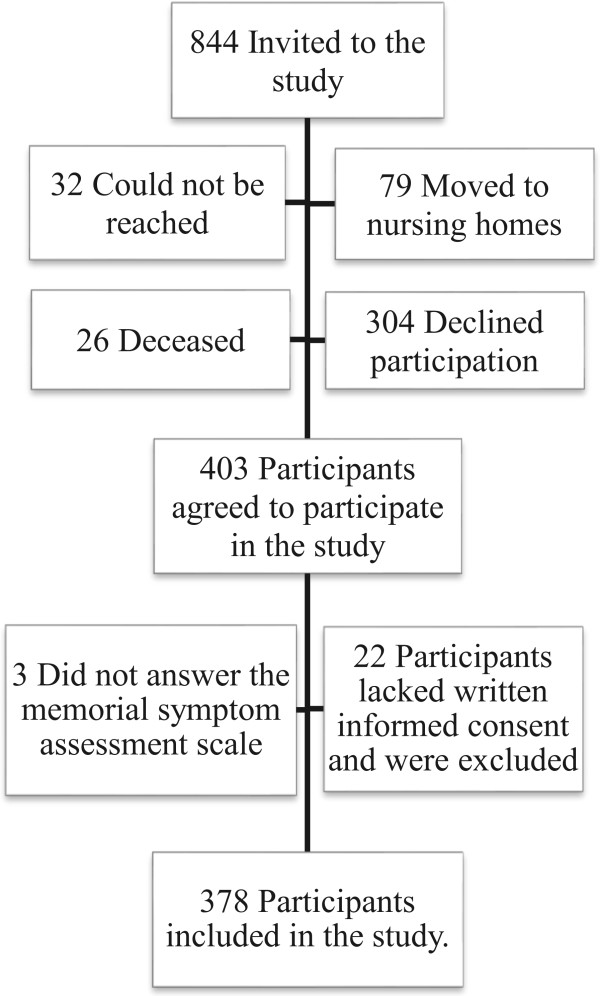
**Flow chart of the inclusion and exclusion process of participants.**

Participants were recruited from one municipality through the patient administrative system of the County Council. Norrkoping is a middle-sized city, (120 000) in the south-east of Sweden, where approximately 9% of the inhabitants are ≥75 years. Inclusion criteria were: people ≥ 75 years, who had been hospitalized ≥ 3 times during the previous year, who had ≥ 3 diagnoses in their medical records according to the International classification of diseases (ICD-10) [18] and who lived at home. The only exclusion criterion was if participants were already living in a nursing home. Eight hundred forty-four older people were invited to participate; of those, 79 were excluded since they had moved to a nursing home just before the study started, 26 were deceased, 32 could not be reached, and 304 declined to participate. Out of the 403 that were willing to participate, in 22 cases written informed consent was missing and they were therefore excluded. In the present study, only those who had completed the symptom assessment instrument were included; in total 378 eligible participants. The flow-chart of the study is visualized in Figure 1.

### Procedures

An invitation letter explaining the purpose of the study was sent to the eligible older people. They were then contacted by telephone, and those who gave oral informed consent were scheduled for an appointment at their home for protocolled directed interviews during which written consent was obtained. All data were collected with protocol-guided interviews, performed by specially trained registered nurses or occupational therapists between February 2011 and December 2011. The study followed the ethical guidelines given in the declaration of Helsinki and was approved by the regional ethical review board in Linkoping (Dnr. 2011/41-31).

### Assessments

#### Symptoms

Symptom prevalence, symptom experience and symptom burden were assessed using the Memorial Symptom Assessment Scale (MSAS) [15]. MSAS includes a large number of commonly occurring symptoms and assesses the prevalence of 32 symptoms. It considers symptoms experienced during the preceding week in terms of three dimensions - frequency, severity and distress - for 24 symptoms, and two dimensions - severity and distress - for eight symptoms. The format for responses is as follows. The prevalence of each symptom is answered by yes/no: frequency on a four-point scale - rarely, occasionally, frequently or almost constantly; severity on a four-point scale - slight, moderate, severe, or very severe; and distress on a five-point scale - not at all, a little bit, somewhat, quite a bit or very much [26]. For clarity of reporting, we used the terms ‘high frequency’, ‘high severity’ and ‘high distress’ for the two highest scores of each symptom dimension. One item dealing with sexual interest or activity was excluded from the original instrument prior to the data collection. The data collector did not feel comfortable asking the old and often lonely people a question about sex. This left the instrument with 31 symptoms. The coding of the instrument was based upon the instruction of the original authors [26]. The symptom burden score was calculated as a mean score of frequency, severity and distress of each symptom [15]. The total symptom burden score per patient (TMSAS) is the mean of all 31 symptom burden scores [26]. The MSAS was originally conducted to assess symptoms in people with cancer [26] but has since been tested and evaluated on people with different chronic diseases [20, 21, 27]. The MSAS is validated and has been used in a Swedish context [28, 29]. The Cronbach’s alpha coefficient for TMSAS in this study was 0.82.

#### Background characteristics

Background data collected in this study included age, gender, current marital status, living situation, next of kin, education level, use of tobacco and alcohol, problems with vision, hearing, and body mass index (BMI). Cognitive decline was measured by the Mini-Mental State Examination (MMSE), which is a validated 30-point brief questionnaire test that is used to screen for cognitive impairment. In this test, 24 points or more is considered as normal cognitive function, 18–23 is mild–moderate cognitive dysfunction, and <18 indicates severe cognitive dysfunction. The likelihood of depression was assessed by using the Geriatric Depression Scale (GDS-15) [30], which is a validated self-reported instrument. Data on the participants’ medical diagnoses were provided by the ‘Data Care Warehouse’, which is a population-based, administrative database run by the County Council.

### Statistical analysis

The statistical analysis is described by frequencies and percentages for categorical variables, and continuous data by means and standard deviations (SD) for normally distributed data. To be able to compare results from this study with previous research, MSAS symptom burden scores are presented as mean (SD) even though the variables were often skewed. A multiple linear regression analysis was performed to determine independent associations between TMSAS and the background characteristics from Table 1. The multiple model was built by entering those variables that had univariate statistical significance with a p < 0.05 in the correlation, retaining those variables with p < 0.05 in the final regression model. All two-way interactions were tested in the model. Data were analyzed using PASW Statistical (SPSS) version 20. The significance level was set at p ≤ 0.05.

**Table 1:** **Background characteristics**

	N = 378
Age (yrs.), mean (SD)	82 (4.8)
Women n (%)	182 (48)
Lived alone n (%)	193 (51)
Elementary school n (%)	305 (81)
Secondary school or higher education n (%)	68 (18)
Poor hearing with or without hearing device. n (%)	130 (34)
Poor vision with or without glasses. n (%)	104 (28)
Smokers n (%)	32 (8)
Alcohol on daily basis. n (%)	27 (7)
BMI (kg/m^2^) mean (SD)	26.1 (4.6)
Underweight (BMI < 18.5) n (%)	8 (2)
Normal (BMI 18.5 ≤ 24.9) n (%)	145 (38)
Overweight (BMI 25 ≤ 29.9) n (%)	140 (37)
Obese (BMI > 30) n (%)	60 (16)
MMSE mean (SD)	26.3 (3.2)
MMSE 20–24 n (%)	65 (17)
MMSE 10-19n (%)	12 (3)
MMSE ≤ 9 n (%)	0
GDS mean (SD)	3.7 (3.0)
GDS ≥5 n (%)	120 (32)
Diagnosis according to ICD 10 Chapter	
01. Certain infectious and parasitic diseases (A00-B99) n (%)	166 (44)
02. Neoplasma (C00-D48) n (%)	158 (42)
03. Diseases of the blood and blood-forming organs and certain disorders involving the immune mechanism (D50-D89) n (%)	116 (31)
04. Endocrine, nutritional and metabolic diseases (E00-E90) n (%)	187 (50)
05. Mental and behavioral disorders (F00-F99) n (%)	127 (33)
06. Diseases of the nervous system (G00-G99) n (%)	130 (34)
07. Diseases of the eye and adnexa (H00-H59) n (%)	219 (58)
08. Diseases of the ear and mastoid process (H60-H95) n (%)	142 (37)
09. Diseases of the circulatory system (I00-I99) n (%)	362 (96)
10. Diseases of the respiratory system (J00-J99) n (%)	210 (55)
11. Diseases of the digestive system (K00-K93) n (%)	206 (54)
12. Diseases of the skin and subcutaneous tissue (L00-L99) n (%)	164 (43)
13. Diseases of the musculoskeletal system and connective tissue (M00-M99) n (%)	295 (78)

*Abbreviations*: *BMI* Body Mass Index, *MMSE* Mini-Mental State Examination, *GDS-15* Geriatric Depression Scale, *ICD* International Classification of Diseases.

## Results

The mean age of the participants was 82 (4.8) years, with an almost equal distribution of men (52%) and women (48%) (Table 1). Significant differences were found both regarding gender and age between people who agreed to participate (n = 381) and those who declined, could not be reached, had moved to nursing home, did not provide informed consent or had recently died (n = 461). People who agreed to participate were slightly younger than the non-participants 82 (4.8) vs.83 (5.4) years (p = 0.024) and more men than women accepted the invitation to participate (p = 0.039).

In total, 51% of participants lived alone and 81% had an educational level consisting of elementary school. Approximately one-third (28%) reported problems with vision, with or without glasses and (34%) reported problems with hearing, with or without hearing device. In total, 8% currently smoked and 7% drank alcohol on a daily basis. The mean score on the MMSE was 26 (3.2), and 20% of the participants had a score below 24 that could indicate cognitive dysfunction. With regard to depression we found that 32% scored equal or higher than 5 on the GDS, indicating the likelihood of depression (Table 1).

Participants in this study had three or more medical diagnoses in their medical record. Almost all participants (96%) had at least one disease listed in ICD chapter 9, diseases of the circulatory system. Participants also had diseases from ICD chapter 9 relating to the musculoskeletal system (83%), diseases of the digestive system (60%) and diseases of the eye and adnexa (58%) (Table 1).

### Symptom prevalence

The mean number of symptoms per patient (total prevalence) was 8.5 (4.6), 30% of the participants reported 10 co-occurring symptoms or more, and three participants (0.8%) reported no symptoms at all. In the total group of respondents, pain was the symptom with the highest prevalence (67%). Half of the participants (47-51%) reported dry mouth, lack of energy, numbness and tingling in the hands and feet. Almost four out of ten (36-42%) experienced problems with feeling drowsy, dizziness, shortness of breath, difficulty sleeping, feeling sad, and worrying (Table 2).

**Table 2:** **Symptom prevalence and symptom experience in older people with multi-morbidity**

		Symptom experience		
Symptoms	Prevalence n/(%)	High frequency n/(%) ^a^	High severity. n/(%) ^b^	High distress n/(%) ^c^
N 378 (%)				
Pain	253 (67)	164 (43)	117 (31)	132 (35)
Dry mouth	193 (51)	103 (27)	55 (15)	49 (13)
Lack of energy	189 (50)	122 (32)	81 (21)	94 (25)
Numbness/tingling in hands/feet	178 (47)	95 (25)	57 (15)	57 (15)
Feeling drowsy	158 (42)	52 (14)	34 (9)	38 (10)
Dizziness	156 (41)	65 (17)	59 (16)	61 (16)
Difficulty sleeping	153 (40)	92 (24)	64 (17)	56 (15)
Shortness of breath	149 (39)	68 (18)	59 (16)	55 (15)
Feeling sad	138 (36)	46 (12)	54 (14)	48 (13)
Worrying	136 (36)	38 (10)	45 (12)	39 (10)
Swelling of arms or legs	130 (35)	N/A	32 (8)	30 (8)
Cough	129 (34)	43 (11)	31 (8)	34 (9)
Itching	118 (31)	48 (13)	38 (10)	32 (8)
Problems with urination	104 (28)	71 (19)	43 (11)	44 (12)
Feeling nervous	103 (27)	36 (10)	28 (7)	31 (8)
Changes in skin	102 (27)	N/A	13 (3)	18 (5)
Feeling bloated	92 (24)	45 (12)	37 (10)	34 (9)
Feeling irritable	85 (23)	25 (7)	21 (6)	18 (5)
Constipation	79 (21)	N/A	33 (9)	30 (8)
Difficulty concentrating	74 (20)	21 (6)	23 (6)	25 (7)
Sweating	71 (19)	26 (7)	19 (5)	19 (5)
Lack of appetite	69 (18)	43 (11)	22 (6)	17 (4)
Diarrhea	60 (16)	22 (6)	34 (9)	30 (8)
Difficulty swallowing	54 (14)	31 (8)	25 (7)	24 (6)
Nausea	52 (14)	14 (4)	17 (5)	17 (5)
Change in the way food tastes	43 (11)	N/A	10 (3)	8 (2)
Mouth sores	42 (11)	N/A	14 (4)	14 (4)
Weight loss	28 (7)	N/A	7 (2)	7 (2)
"I don’t look like myself"	23 (6)	N/A	10 (3)	11 (3)
Hair loss	19 (5)	N/A	6 (2)	6 (2)
Vomiting	14 (4)	1 (0,3)	5 (1)	5 (1)

^a^Percentage of people with the symptom reporting a high frequency = "frequently" or "almost constantly." ^b^Percentage of people with symptom reporting high severity = "severe" or "very severe." ^c^Percentage of people with the symptom reporting a high distress "quite a bit" or "very much." N/A = Not Applicable.

### Symptom experience

Pain was the symptom reported with the highest frequency score, with four out of 10 participants (43%) reporting that the symptom had occurred frequently or almost constantly during the preceding week. Nausea and vomiting were reported by less than 5% (Table 2).

Pain was a symptom that most participants reported as severe. One-third (31%) of the participants gave the symptom a high severity score (severe or very severe). Difficulty sleeping and lack of energy were reported by 17-21% respectively as being "severe or very severe." (Table 2).

Pain and a lack of energy were reported with a high symptom distress score (quite a bit or very much distress) by 35% and 25% of the respondents respectively. Numbness and tingling in the hands and feet, dizziness, shortness of breath and difficulty sleeping were reported by 14–16% of the participants as causing high symptom distress (Table 2).

Symptom burden is reported for participants who experienced the symptoms during the preceding week and not for the total group of 378 participants. For 29 of 31 assessed symptoms, patients who reported the respective symptom had a mean score of ≥ 2.0, and seven symptoms were reported to have a mean symptom burden of more than 2.6 (Table 3). Pain had the highest symptom burden score; the 248 patients who reported having pain had a mean symptom burden of 2.8 (0.68). With regard to pain we found that 45% of the 248 participants who reported pain had a symptom burden score of ≥ 3.0 (Table 3).

**Table 3:** **MSAS symptom burden score of older people who reported the symptom as present during the previous week**

Symptom	Number of patients who reported the symptom	Symptom burden score	(SD)
Pain	248	2.8	(0.68)
Lack of energy	191	2.7	(0.67)
Difficulty swallowing	53	2.7	(0.73)
"I don’t look like myself"	22	2.7	(1.03)
Difficulty sleeping	152	2.6	(0.70)
Problems with urination	103	2.6	(0.76)
Diarrhea	59	2.6	(0.78)
Numbness/tingling in hands/feet	175	2.5	(0.74)
Shortness of breath	146	2.5	(0.67)
Feeling sad	135	2.5	(0.72)
Feeling bloated	90	2.5	(0.65)
Constipation	79	2.5	(0.72)
Dry mouth	189	2.4	(0.74)
Dizziness	151	2.4	(0.79)
Worrying	135	2.4	(0.64)
Feeling nervous	102	2.4	(0.72)
Difficulty concentrating	75	2.4	(0.67)
Lack of appetite	67	2.4	(0.67)
Itching	114	2.3	(0.76)
Sweating	67	2.3	(0.80)
Mouth sores	41	2.3	(0.87)
Feeling drowsy	151	2.2	(0.66)
Cough	125	2.2	(0.66)
Nausea	51	2.2	(0.62)
Hair loss	19	2.2	(0.91)
Vomiting	14	2.2	(0.32)
Swelling of arms or legs	126	2.1	(0.80)
Feeling irritable	82	2.1	(0.72)
Change in the way food tastes	41	2.1	(0.76)
Weight loss	24	1.9	(0.97)
Changes in skin	97	1.8	(0.72)

The MSAS symptom burden score is the mean score of the three dimensions: frequency, severity and distress.

### Factors related to symptom burden

For the total group of participants the total symptom burden (TMSAS) had a mean (SD) score of 0.62 (0.41). TMSAS was significantly correlated with sex (r_s_ = -0.16), with women reporting a higher score than men. TMSAS was also significantly correlated vision (r_s_ = -0.14) and hearing (r_s_ = -0.10), with participants with poor vision or hearing reporting higher TMSAS scores. TMSAS was correlated with risk of depression (r_s_ = 0.57), diseases of mental and behavioral disorder (r_s_ = 0.21), diseases of the digestive system (r_s_ = 0.16) and diseases of the nervous system (r_s_ = 0.11). In the multiple linear regression, TMSAS was independently related to poor vision (beta = -0.153), risk of depression (beta = 0.566), and diseases of the digestive system (beta = 0.109). The *R*^*2*^ for this model was 0.38, indicating that 38% of the variance of TMSAS could be explained by these three predictors (Table 4).

**Table 4:** **Dependent Variable TMSAS N = 378**

Independent variables	Unstandardized β	Standardized beta	P level
Poor vision	- 0.140	-0.153	<0.001
Likelihood of depression	0.077	0.566	<0.001
Diseases of the digestive system	0.090	0.109	0.008

Multiple linear regression analysis of the dependent TMSAS and the independent poor vision, likelihood of depression, diseases of the digestive system in 378 older people with multimorbidity.

R square 0.38 P < 0.05.

*Abbreviation*: *TMSAS* Total Memorial symptom assessment scale.

Poor vision = self-reported, Likelihood of depression = Geriatric Depression Scale ≥5, Diseases of the digestive system = participants with ICD (K00-K93) in their medical record.

## Discussion

There is a lack of knowledge concerning the multidimensional symptom profile and symptom burden in older people with multimorbidity. An important finding of this study was that older community-dwelling people with multimorbidity suffer a considerable symptom burden, with a mean of eight symptoms per person, and some people even reporting 10 symptoms. Almost seven out of ten older people suffered from pain. Pain was also the symptom with the highest frequency, severity, and distress and had the highest symptom burden score of all 31 symptoms. Three factors were found to be independently associated with total symptom burden: poor vision, likelihood of depression, and diseases of the digestive system.

Almost seven out of 10 participants in this study had experienced pain during the preceding week, which is a high number but in line with some other studies in which community-dwelling older people reported a pain prevalence of between 20 to 79% [31, 32]. As reflected by the high symptom burden, our findings show that pain causes older people a considerable amount of suffering. This group of older people living in a home had a burden of pain that was comparable to the scores of hospitalized cancer patients near the end of life [27], which was not something we had expected. Earlier studies described pain as underdiagnosed and undertreated [31, 33] and found that, irrespective of clinical diagnosis, 25% of older people do not receive analgesic treatment for pain, and people older than 85 are even less likely to receive it [33]. At the same time, polypharmacy is a huge problem within the group of older people with multimorbidity; inappropriate drug intake causes complications and symptoms, leading to repeated visits to the emergency department and hospitalization [34]. There has been a lack of age-specific standardized management guidelines for geriatric pain, and health professionals have felt that multimorbidity complicates appropriate management [35]. Just recently, new guidance for treating pain in the elderly was published [31].

In addition to pain, lack of energy, difficulty swallowing, "I don't look like myself", difficulty sleeping, problems with urination, and diarrhea had a high symptom burden in those who experienced them. These symptoms seem rather non-specific, that is, not related to one specific medical diagnosis, and are not always recognized as being important. However, earlier studies have shown that older people with a high symptom burden have a poorer quality of life and higher use of health services [18, 19]. To facilitate and improve symptom management, health care providers should not only look at and assess disease-specific symptoms but also take general symptoms into consideration.

We also found that poor vision, likelihood of depression, and diseases of the digestive system were associated with total symptom burden. It is known that people in a depressive mood experience a higher symptom burden than those who are not [36]. Earlier studies have confirmed the association between depression and pain [37]. If recognized and treated, depression is often reversible, but if left untreated, depression may result in the onset of physical, cognitive, functional, and social impairment, as well as decreased quality of life, [38] and higher mortality [39]. The independent relationship between poor vision and the total symptom burden scale has not previously been described, but other studies have reported that people with poor hearing or vision are more likely to experience disability, and that there are associations between poor vision, poor hearing and depression [40]. Earlier studies have shown that people with diseases of the digestive system have a lower health-related quality of life [41, 42] and that the severity of the gastro-intestinal symptoms and health-related quality of life are associated [41]. Reflecting on the high symptom burden in multimorbid older people, we observe that in our current health care system most care is organized from a single disease perspective, an arrangement into which older people with multimorbidity do not quite seem to fit [6]. These older people have reported feeling unwelcome or even like a burden when they need to seek care [43]. However, health care providers still have the responsibility to give patients optimal support to restore or at least achieve an acceptable level of symptom relief [44]. A routine of a broad assessment of symptoms and symptom burden (not restricted to the disease-specific burden) might lead to better symptom management [19, 45] which could maintain independence and functional ability, and sustain or improve quality of life for older people with multimorbidity.

### Strengths and limitations

The strength of this study is that all data are assessed by protocol-guided interviews, leaving us with little missing data. However, there is still a risk that the older people’s symptom experience or burden have been underestimated since studies have shown that when questionnaires are self-administered instead of interviewer-administered the scores tend to be higher, so this should be taken into consideration when the scores are interpreted [46, 47]. We also realize that this is a rather small cross-sectional study, with participants recruited from only one city and with a higher percentage of men than in the general population [48]. The result of this study cannot be generalized to all community-dwelling older people, since they represent a unique group with many diseases. Nevertheless, the result ought to be generalizable to groups with similar conditions and living in a similar context. Another limitation of this study is the lack of data on diseases from all the ICD chapters. Data on diseases would have made it possible to identify disease clusters and use these to predict TMSAS or to describe the symptom experience of specific clusters. However, we still believe that although clustering of diseases is important and an area of interest for future studies, our results bring forward the unique perspective of an elderly population with several diseases living with a high symptom burden.

## Conclusion

A large proportion of the older people with multimorbidity living in the community suffer from a high symptom burden and a high prevalence of pain. These people have an unmet need for optimized treatment focusing on the assessment, management and maintenance of the total symptom burden. People with poor vision, likelihood of depression and diseases of the digestive system are at risk of facing a higher symptom burden and might need age-specific standardized guidelines for appropriate management.

## Abbreviations

BMI: Body mass index
MMSE: Mini-mental state examination
GDS-15: Geriatric depression scale
ICD: International classification of diseases.
